# A Critical Examination of the Use of Trained Health Coaches to Decrease the Metabolic Syndrome for Participants of a Community-Based Diabetes Prevention and Management Program

**DOI:** 10.4172/2472-1654.100038

**Published:** 2016-11-01

**Authors:** Brandon Lucke-Wold, Samantha Shawley, John Spencer Ingels, Jonathan Stewart, Ranjita Misra

**Affiliations:** West Virginia University, Morgantown, West Virginia, USA

**Keywords:** Community Based Participatory Research (CBPR), Metabolic syndrome, Diabetes Prevention and Management, Obesity, Lifestyle medication, Cardiovascular disease, Partnerships, Health coaches, Health outcomes

## Abstract

The epidemic of obesity and diabetes in the United States poses major challenge to the prevention and management of chronic diseases. Furthermore, when this is viewed in other components of the metabolic syndrome (i.e., the burden of high cholesterol and hypertension), the prevalence of the metabolic syndrome continues to rise in the USA continued challenge is how to deal with this epidemic from a medical and public health standpoint. Community Based Participatory Research (CBPR) is a unique approach and offers a novel perspective for answering this challenge. A critical set of goals for CBPR is to address health disparities and social inequalities while getting community members engaged in all aspects of the research process. Utilizing the West Virginia Diabetes Prevention and Management Program and trained Health Coaches as a model, we discuss topics of consideration related to CBPR, involving trained health coaches, optimizing early adoption of healthy lifestyle behaviors, and enhancing participation. Through careful project planning and design, questions regarding disparities increasing susceptibility and preventive efforts within the community can be addressed successfully. These topics are part of a broader integration of theories such as participatory research, community engagement, and outcomes measurement. The understanding of the pathophysiology and epidemiology of the metabolic syndrome can help frame an appropriate strategy for establishing long-term community-wide changes that promote health. In order to continue to improve investigations for preventing the metabolic syndrome, it will be necessary to have aggressive efforts at the individual and population level for developing culturally sensitive programs that start early and are sustainable in practical environments such as the workplace. In this comprehensive review, we will discuss practical considerations related to project design, implementation, and how to measure effectiveness in regards to reducing the burden of the metabolic syndrome.

## Introduction

Type II diabetes is increasing worldwide with upwards of 58% of patients with diabetes developing the metabolic syndrome [1]. Early interventions and screening in individuals with pre-diabetes has been shown to decrease the risk of developing diabetes [2]. Only recently have outcomes such as the metabolic syndrome, cardiovascular disease, and obesity been reported following participation in diabetes prevention and management program [3]. According to the National Cholesterol Program Adult Treatment Panel the metabolic syndrome consists of having 3 of the following: Abdominal obesity, dyslipidemia, hypertension, insulin resistance, or increased risk for thrombosis. The metabolic syndrome significantly increases the risk for cardiovascular events such as stroke and myocardial infarction [4]. A recent meta-analysis confirmed however that diabetes prevention and management programs emphasizing community engagement and the tracking of food consumption were more successful than medications in reducing the metabolic syndrome and adverse cardiovascular events [5].

In this review, we highlight our own experiences with a community-based program for diabetes prevention and management that focuses on participatory research, community engagement, and careful monitoring of clinical outcomes. Using this program as a reference, important principles are discussed including the selection site of the study, the importance of participant feedback, successful implementation of health coaches, and how to optimize the adoption of the interventions. These principle components will be addressed under the broader framework of lifestyle adjustments that were designed to decrease the risk associated with the metabolic syndrome, and associated diabetes and CVD risk. To understand how these interventions are intended to function within the community, we will briefly review the pathophysiology and epidemiology that contributes to the metabolic syndrome. Finally, future advancements will be suggested for the diabetes prevention and management program with the intention of improving avenues that reduce the burden of the metabolic syndrome, diabetes, and cardiovascular disease within at-risk communities.

## Principle Components

### Metabolic syndrome pathophysiology

Insulin resistance is the underlying pathophysiological process defining the metabolic syndrome [6]. Recent evidence suggests that overactive glucocorticoid signaling could be contributing to this resistance [7]. The mechanism is mediated potentially through an increase in adipose tissue, which contributes to the release of adipokines [8]. Adipokines lead to a chronic proinflammatory state that can damage pancreatic cells and lead to insulin insensitivity due to overactivity [9]. Glucocorticoid signaling is associated with increased life stress and often begins early in childhood [10]. Stress in childhood can increase cardiometabolic factors and increase adipokines predisposing individuals to the metabolic syndrome later in life [11].

Furthermore, high stress exacerbates the risk for getting the metabolic syndrome among adults. Recently, a longitudinal study found that increased work-related stress had negative effects on four of the five metabolic syndrome diagnostic criteria: Blood pressure, HDL cholesterol, lipid profiles, and fasting blood glucose. Participants with work-related stress had 2.68 the odds for developing the metabolic syndrome compared to non-stressed controls [12,13]. In addition to upregulating adipokines, work related stress might decrease IL-18, which has been shown to be a contributory factor to the development of obesity [14]. The Whitehall II study found that workers of lower socioeconomic status were at increased risk of becoming obese compared to workers of higher socioeconomic status with the same reported stress level [14]. This indicates that health disparities may be present and are dependent on the perceived environmental control of an individual. This perceived control could directly regulate underlying physiologic responses and increase physical susceptibility to stress.

### Metabolic syndrome epidemiology

**Figure 1:**
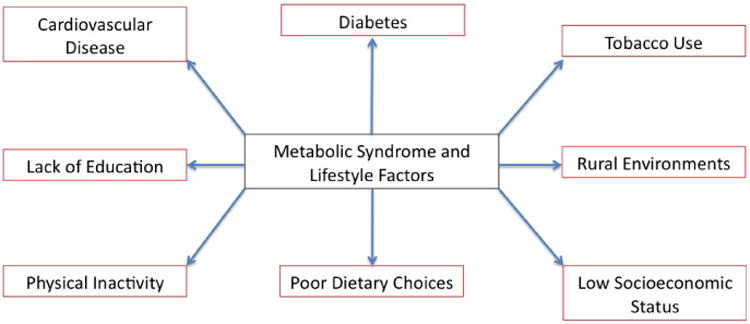
Metabolic syndrome is associated with multiple lifestyle factors.

Approximately 25% of United States adults have been diagnosed with the metabolic syndrome [15]. Insulin resistance has been proposed as the leading risk factor for developing the metabolic syndrome [16]. Patients with the metabolic syndrome and diabetes have the highest prevalence of coronary heart disease at 19.2% [17]. Coronary heart disease is directly tied to vascular events such as stroke and myocardial infarction, and is the leading cause of death in the United States. Associated with these vascular events are unhealthy behaviors such as tobacco use, physical inactivity [18], and poor dietary choices (Figure 1).

Interventions that target a change in lifestyle habits thereby offer the best solution for reducing the risk factors via dietary, lifestyle and behavior modifications as a means of reducing the likelihood of developing metabolic syndrome, as associated risk for diabetes, obesity, hypertension and cardiovascular disease. The primary dilemma is addressing the gap between available knowledge and effectively applying that knowledge to lower the risk. A chronic disease such as diabetes, metabolic syndrome, and cardiovascular disease predominately affects those in the lowest social economic class and rural communities [19]. Women in the lowest income bracket have a 1.81 odds ratio of developing the metabolic syndrome compared to the general population [20]. Often those in rural areas with poor access to care also are at increased risk for health disparities. West Virginia is one of the poorest states in the nation and has the highest levels of most chronic disease categories such as diabetes (4^th^ in the nation), obesity (1^st^ in the nation), hypertension (2^nd^ in the nation), cardiovascular disease (2^nd^ in the US) and the metabolic syndrome. West Virginia is an Appalachian rural environment that has a culture of independence and historically does not trust outsiders. Additionally, they may not know how to take care of their health due to low education and social economic status. The utilization of the CBPR approach helps participants to “own” and contribute to program success. As Cargo and colleagues emphasize, the primary goal of CBPR is to address health disparities [21]. Designing a program to address the metabolic syndrome and diabetes prevention and management in rural West Virginia falls in line with the health disparity goals and vision of CBPR.

### Program overview

**Figure 2:**
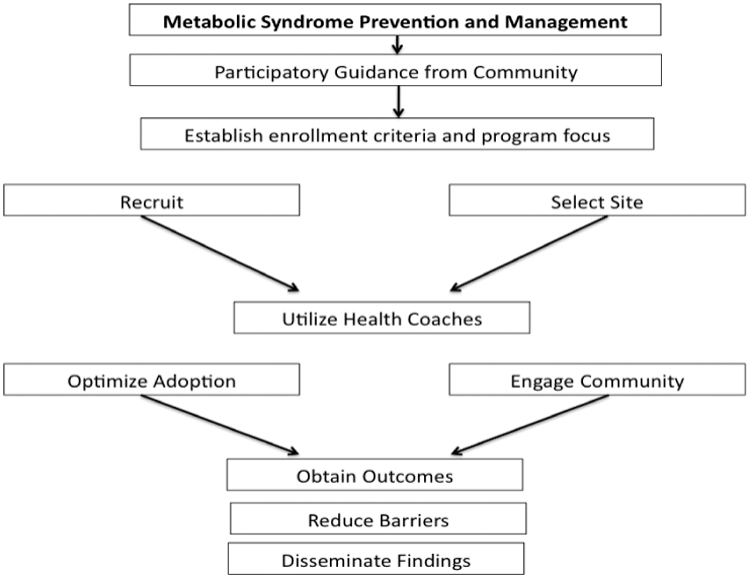
Schematic outlining the flow process for metabolic syndrome prevention and management programs.

The Diabetes Prevention and Management program is an evidence-based program that is being implemented at two-sites: Morgantown and Charleston West Virginia, and it are currently in its second year of implementation. The program consists of twenty-two sessions taught by trained health coaches and community experts. The recruitment target was enrollment of 60 participants into the study for year 2; the study team reached 100% of their recruitment target in October 2015, with 70 participants recruited into the study. Participants voluntarily agreed to participate in the 1-year DPM program. In order to be eligible for the program, participants had to be over 18 years of age and have dysglycemia (i.e., have a diagnosis of diabetes or pre-diabetes). The participants were eager to engage in a program with a focus on non-pharmaceutical lifestyle modification through education and group support. Data is being collected on multiple factors including demographics, anthropometrics, clinical outcomes, satisfaction with the program, and calorie consumption as well as step tracking. In this review, we will use the ongoing program as a basis for evaluating implementation strategies to address the metabolic syndrome (Figure 2).

### Selecting a site

One of the most critical components for CBPR is selecting appropriate community partners and sites [22]. Partners can contribute financially, donate time, or offer resources such as use of a building or equipment for sessions. Commonly used sites include community centers, local clubs, churches, or other place of worship pertinent to Christians, Muslims, Jews, or Hindus etc. These buildings are designed to host large gatherings. By having the meeting in the community, participants will likely feel more comfortable and view the location as neutral because it is outside of the university setting. A university setting could reduce participation due to distrust of research centers, anxiety of getting to the sessions due to confusing building layouts, parking issues, and early morning or late evening times for hosting sessions. Many participants for diabetes programs also receive care from hospitals and academic centers making the location less than ideal for implementing a new community-based intervention. Academic centers that offer care in conjunction with programs are often associated with increased participant anxiety [23]. Places of worship such as churches, synagogue, mosques, and temples offer a safe environment in multiple diverse communities and are traditionally in convenient locations within the community to host events. Hosting events in a place of worship has been shown to be ideal for reaching large participant audiences and helping solve health disparities [24]. Based on these considerations, a church-based community site for the Diabetes Prevention and Management program in West Virginia (96% of the population is Non-Hispanic White and Christians) is used to provide an example of potential implementation facilitators and challenges.

Churches in West Virginia and other places of worship nationally often offer unique advantages in that many have an attached kitchen for cooking demonstrations and space for physical activity demonstrations. This is highly important for programs dealing with prevention of chronic diseases such as diabetes, obesity, metabolic syndrome etc. Prevention programs that show demonstrations of healthy food preparation and physical activity are more effective in changing lifestyle habits than programs without these demonstrations [25]. Many diabetes prevention programs include tracking calories, which is often confusing for participants. Demonstrations allow for better adaptability of the proposed intervention and can improve the ability of participants to improve tracking the calories in a meal [26]. By developing effective partnerships with religious organizational leadership and their board members, the resources at these places of worships can be readily used for implementation of the program. Another advantage of using such a site is the ready availability of parking. Morris found that in rural community's site accessibility including ease of parking was vitally important for continued participation throughout the program [27]. Having a readily accessible site helps encourage participation even when weather is a potential barrier.

### Participatory feedback

Once the site was selected and the program is underway, it is important to garner feedback from participants. The key to a successful diabetes prevention and management program is ensuring sustainability. Focus groups offer an interesting and valuable means by which feedback can be obtained. A CBPR program for diabetes prevention in Harlem used mid-program focus group surveys to help restructure the second half of the program [28]. Similarly, the DPM program used focus groups to assess process related improvements for the second year. Additionally, feedback from the first year health coaches was used to improve the training and programmatic activities to address diabetes and metabolic syndrome disparities in two church sites for the diabetes prevention and management program, located at opposite ends of the state (∼200 miles apart).

A local community champion was selected for the site located 3 hrs away from the university. The community champion is a local female student and a health coach who grew up in the region and works for the state department. Adeniyi et al. found that enlisting the help of community champions was essential for decreasing risk factors for the metabolic syndrome [29]. Community champions are the “go to” person when questions arise about the program or when the participants seek additional support or motivation. The type of community champion described above is specifically termed a project champion. Project champions address the concerns of individual participants instead of the needs of a health system [30]. The second type of champion is the systems champion. In this case, the researcher with expertise in diabetes fulfills this role. The systems champion makes sure the program runs smoothly, that the technical questions of participants are answered, and that sustainability is emphasized. The key is constant dialogue between participants and continual interaction between both types of community champions. The iterative process is important in eliciting lasting and meaningful change for participants and reducing the burdens of the chronic diseases through prevention [22].

### Health coaches

The health coaches attended 20 hrs of training using a curriculum developed by the program team; undergraduate, master and doctoral students enrolled in professional programs and other majors (Public Health, Nursing, Medicine), College of Physical Activity and Sports Sciences, Exercise Physiology, Human Nutrition and Department of Communication. The health coaches were provided opportunity to participate in the planning, participant recruitment, and screening of participants.

For preventing chronic diseases and syndromes such as the one focused for this paper (metabolic syndrome), healthy lifestyle changes depend on three important components: Change in mindset, change in physical activity, and change in eating habits. A primary way to encourage these changes is through education. Health coaches trained in diabetes education have been shown to be highly effective [31,32]. Sessions that are run by trained health coaches have been associated with reduced hemoglobin A1c and LDL levels as well as reduced weight through the course of the intervention series [33]. Health coaches are viewed as peers instead of researchers, which facilitates increased trust during interactions [34]. The process of change is gradual and often involves small increments of progress. To facilitate healthy reminders, health coaches reach out to participants' in-between sessions to check-in and encourage adjustments in lifestyle habits.

The use of advanced technology such as texting and email makes the feasibility of contact between coaches and participants efficient and productive. Texting has emerged as an effective way for coaches to provide positive feedback and important reminders to participants [35]. In addition to direct contact, sessions are video recorded and posted on a closed YouTube link for direct viewing by those who missed the session. This dualistic strategy combines direct contact through in-person sessions with distant learning through advanced media and communication with health coaches. Distance learning is emerging as a useful supplemental tool for enhancing early adoption of innovative changes by participants [36]. Because chronic diseases develop through a combination of fatalistic thinking, inactivity, and poor lifestyle choices, the ability to provide reminders for small but manageable changes throughout the week is highly valuable.

In order to help participants' stay on track, calorie-tracking books and pedometer step counts were used in addition to weigh-ins at each session. Participants were encouraged to record their daily food intake by hand in the food logs provided. They were instructed to be honest, accurate, and complete it each week to build awareness of their eating behaviors. Food logs and dairies increase awareness of extra calories and have been shown to be a powerful tool in prior research [37]. The health coach provided valuable feedback to the participants at the end of each week by writing notes in the completed tracking books. Although calorie tracking is a known barrier, feedback by health coaches helped encourage completion and supported weight loss by participants. In the lower Mississippi delta, calorie tracking with feedback was associated with a 2.7% drop in body weight over a 16-week course [38]. Pedometer use also has a proven track record. In Japan, high frequency calls by health coaches significantly increased participant step counts and prevented the transition to diabetes in pre-diabetic participants [39]. Health coaches play a valuable role in providing support for participants and encouraging a change in mindset. By addressing the participant concerns, lasting changes can be implemented that extend well beyond the completion of the program.

### Optimizing adoption

A key component for participant adoption is to move CBPR from the margins to mainstream [40]. One of the ways this is done is by making the environment comfortable for participation. Participants are more likely to join a program if the research team is friendly and the program has a proven track record. Retaining participants throughout the course of an intervention is essential for establishing sustainable behavioral change. Westfall urges that participatory research requires community members to work together with researchers to make lasting behavioral changes through the process of education [41]. In the DPM program, participants obtain change through weight loss and reducing hemoglobin A1c levels.

The educational component provides valuable support and dissemination of ideas that allow participants to incorporate new habits. In addition to improving participant's knowledge of healthy lifestyle, other important educational pieces focused upon dealing with small setbacks, coping with stress, and how to get back on track after a perceived slip up. To address these hurdles, the systematic approach of consistent sessions with weekly feedback has been shown to be more effective than single interventions or unconventional approaches [42]. Since the participant group was sufficiently large (20 to 40 participants in each site), early adopters of the intervention helped infuse the desire to change by engaging other members in meaningful interactions and peer-to-peer encouragement with personal stories of weight loss and tools that worked for them. Over time more and more participants will begin to implement the changes modeled by peers [43].

Maintaining a healthy diet and increasing physical activity will reduce the prevalence of metabolic syndrome by decreasing risk factors, such as diabetes and obesity. Many participants are knowledgeable of individual conditions such as increased blood pressure, blood sugar, and abnormal cholesterol, but have trouble understanding how the clustering of these factors increases the risk for cardiovascular disease. The more important struggle is that participants have to implement their knowledge into behavioral modifications. Green and colleagues argue that although knowledge is present, it is not readily being implemented into preventive programs by public health researchers [44]. Therefore, it is important for researchers to engage in CBPR. CBPR helps transition knowledge into practice [45]. It requires feedback from participants, and for researchers to leave the ivy towers of academic centers to integrate into community settings. By using such an approach, the research becomes focused upon assessing and ameliorating health disparity and developing pragmatic community-based and clinical trials instead of their overarching desire for data collection in controlled conditions.

## Approaches

### Participatory research

One of the underlying core principles of participatory research is empowering people for change. Altman and colleagues showed that the combination of education, health screenings, and discussions about changing attitude and perceptions was successful in reducing the cardiovascular risk factors associated with the metabolic syndrome [46]. Interviewing participants about their perceived barriers is highly important for this iterative process. Successful interviews can help pinpoint factors that stifle motivation and inhibit the transformation process [47]. As previously mentioned, effort-reward imbalance in the workplace could be one factor that increases stress and leads to unhealthy behavior and related chronic diseases [48]. Participatory research requires engagement from individuals to design pragmatic solutions that can reduce work place stress.

Collective solutions generated by participants of the DPM program in West Virginia include changing where they eat lunch, avoiding negative co-workers, and establishing better habits in the workplace. One successful action-based habit is parking the car further from the entrance to inspire increased physical activity throughout the day. Survey responses collected at baseline, 6 months, and 1 year will be analyzed and the data used to disseminate knowledge for the implementation of future programs [49]. On a larger scale, the data will be submitted for publication increasing the community and scientific knowledge base. This knowledge base already contains over 200 action-based and goal-oriented strategies for dealing with barriers [50]. Application of these strategies can then be directly correlated to diet change and metabolic outcomes. In Hawaii, action-based strategies significantly reduced the percentage of fat and saturated fat consumption by participants [51].

Another example of a successful participatory approach for metabolic syndrome prevention was pioneered in the Healthy Lifestyle Change Program. They emphasized the significance of increasing knowledge, skills, and self-efficacy. Programs are normally effective at increasing knowledge but potentially lack in the transfer of skills and self-efficacy. By employing peer mentors to help encourage walking and healthy diets, participants in the Healthy Lifestyle Change Program had an average BMI reduction of 0.5 kg/m^2^ [52]. Peer mentors ensure appropriate modeling and can help in obtaining self-efficacy. Mohatt et al. adapted a similar approach and found that tribal community support in Alaska helped to encourage healthy lifestyle, activity, and diet habits [53]. Participatory research is focused on the participant and how decisions shape their habits. By changing decisions, participants engaged with the program and form and sustain new healthy patterns of behavior.

### Community engagement

Community engagement differs in its primary focus. For the lifestyle programs to address chronic diseases and metabolic syndrome, the community engagement approach would emphasize changes that benefit the collective community and not necessarily individual participants. The Vida Sana community engagement program translated the available diabetes and metabolic syndrome prevention literature into Spanish [54]. By providing translation, it allowed for a broader dissemination of knowledge to Hispanic communities. Similarly, Centis et al. established a lifestyle community engagement project that increased the availability of behavior therapy in the community [55].

For the West Virginia Diabetes Prevention and Management program, pre-screenings were utilized at local mall, restaurants, and churches. The pre-screenings increase awareness about diabetes prevention and management, provided potential participants with important health information, and the posted fliers in clinics, hospitals, restaurants, grocery stores, and churches provided a community-wide reminder about improving health. Screening is an example of community engagement especially when it addresses an area of health disparities on a larger scale [56]. Another goal of community engagement is to enhance enrollment from a target audience [57]. The flyer design and description highlighted the target audience of individuals with diabetes or pre-diabetes. A potential barrier is identification of the community members most relevant for the program. Heller and colleagues found that working with health care providers to help identify participants was an effective strategy for maximizing community engagement [58].

A novel strategy for approaching diabetes prevention is to consider social environment, culture, and local values in designing programs [59]. Values prominent in West Virginia include strong family ties, generosity to neighbors, loyalty to state, respect for elders, and appreciation of Appalachian heritage [60]. These unique traits, shared by the West Virginia community, offer a productive approach for designing implementation programs. A diabetes prevention and management design should ideally involve peer-to-peer support and group discussions in order to capitalize on the community wide traits. Evidence suggests that in communities valuing generosity and loyalty, peer-to-peer support is more readily received [61]. In the West Virginia DPM program, group discussions allowed ideas to be shared and evaluated by the whole community of participants.

### Obtaining outcomes

**Figure 3:**
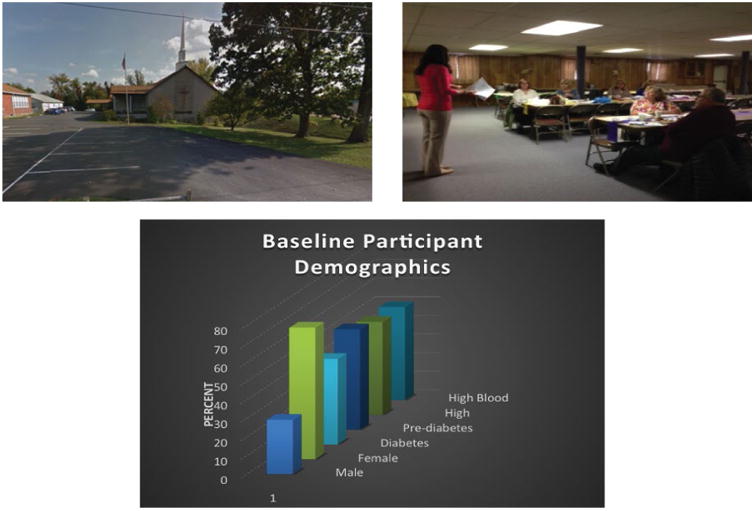
Pictures showing the importance of selecting a site, utilizing health coaches, and collecting relevant outcomes.

Uncontrolled metabolic syndrome can lead to life threatening cardiovascular events, diabetes complications, cognitive decline, and kidney failure [62]. One of the first organs affected by the metabolic syndrome is the kidney. Urinalysis can help identify premature kidney failure and guide treatment [63]. Cardiometabolic tracking can help monitor heart failure and cardiovascular events [64]. It will be important to collaborate and communicate with participants, primary care providers, and specialists to track their metabolic profile over time (Figure 3). Reducing BMI significantly decreases the risk for developing the metabolic syndrome [65]. Hence, feedback to DPM participants about weight change were provided on a weekly basis and assessment of pre- mid- and post-program weight loss provided using reader friendly charts.

In order to track the effectiveness of preventing the metabolic syndrome throughout the course of a program, biometrics and blood work must be collected. Waist circumference has a high correlation with body mass index and can be used as a measure of obesity [66]. Research is still being done to investigate the clusters of metabolic symptoms and which clusters have the greatest risk for a detrimental outcome [67]. It is currently unknown whether clusters of the symptoms making up the metabolic syndrome have variable risk assessments. The one factor that has most consistently been tracked is weight loss due to simplicity and historical controls [68].

Health metrics such as dietary consumption and physical activity level can be correlated to changes in outcomes. Although not a randomized-control trial, the Diabetes Prevention and Management program in West Virginia tracks outcomes from baseline to completion of the program. This allows for careful tracking of metabolic changes at various times throughout the program and correlating changes to the educational intervention. A national consensus meeting in Canada just announced a renewed focus on implementing diabetes prevention programs that track long-term clinical outcomes [69]. Weinhold et al. pioneered such a program tracking long-term outcomes at work. The program was implemented during lunch time in the workplace and participants on average had a 5.5% reduction in BMI and a mean reduction of 8.6 on fasting blood glucose [70]. Workplace programs have obvious benefits for helping target stress induced eating and physical inactivity among white-collar workers.

## Future Directions

### Reducing the burden

The metabolic syndrome is quickly emerging as a global pandemic as both obesity and diabetes increase in prevalence [71]. This syndrome unduly burdens West Virginia and is associated with diabetes and cardiovascular disease and its complications. In order to reduce the burden in this medically impoverished state, it will be necessary to implement preventive programs on a wide scale. One of the primary limitations for preventive programs is a lack of sustainable funding. A recent meta-analysis found that the vast majority of diabetes prevention programs provide cost savings due to improved health compared to medical treatment alone [72]. A limiting factor is that pharmaceutical companies fund a large percentage of diabetes research. This predisposes the field to an unequal distribution of studies looking for novel pharmaceutical treatments with very few looking at prevention.

Woolf argues however that this heavily weighted T1 research focus is limiting translation. T3 research focuses on practice-based research, which has high practicality [73]. Recently the National Institutes of Health has recognized the importance of this type of research and established the clinical translational science awards (CTSA). As Zerhouni points out, CTSAs are an experiment to show the American people a viable return on taxpayer investment [74]. Although in its infancy, new CBPR studies are emerging to address health disparities. Time will tell if a transition occurs from the heavy T1 focus i.e., basic research tested for clinical and/or applicability to more practical T3 research of applying knowledge about how interventions work in real-life world settings. To offset the heavy emphasis on pharmaceutical research, it will be necessary for private foundations to provide additional resources for prevention research.

For the West Virginia Diabetes Prevention and Management program, Rotary International and the Eye Foundation of America were co-sponsors. Rotarians in West Virginia are heavily invested in the Action Group for Diabetes. Eliciting the help and funding support of co-sponsors is essential for sustainability. Israel describes the process of working with co-sponsors as a mutual learning and empowering environment that fosters unique changes that address social inequalities [22]. By using such a community centered funding design, the people of West Virginia actively engage in finding solutions for the problems that burden the state [75]. Over time this pioneering collaboration can be used as an example to pilot other CBPR projects across the state.

### Finding common ground

A key goal of CBPR is participant involvement in all stages of the research process. As data is gathered from the West Virginia Diabetes Prevention and Management program, it will be important to collect qualitative information from participants for inclusion in outgoing manuscripts and reports. Participants should also be offered positions on a community panel to decide appropriate targets of investigation for the community. Through relationship building during the course of the program, it will be possible to recruit and encourage research participants to take an active role in data analysis and program advocacy [76].

Program improvement should be at the forefront of every CBPR study. Several of the first year participants decided to join the cohort for the subsequent year. Through feedback and guidance from these participants, sessions were improved to meet the needs of the community. Israel describes this process as both cyclical and iterative [22]. The program is sustainable through repetition but is also evolving and constantly improving through feedback. As the program grows, it will be important to establish new pilot sites in order to keep the groups relatively small. Small groups that are approximately 30 members allow more focused discussion and peer-to-peer support.

Results regarding study effectiveness must be distributed to a larger audience as a whole. In order to maintain funding, it is necessary to demonstrate that prevention is more cost-effective than treatment. Healthy individuals are better able to contribute to the community through volunteering, work, philanthropy, and social activities. Koh in Vision for Healthy People 2020 proposes that environments must be established where people can contribute to advancing life quality and quantity [77]. Reducing the burden of the metabolic syndrome falls closely in line with this healthy environment. Fortunately, West Virginia has abundant natural green space that can facilitate increased physical activity. National and state parks are in abundance with hiking trails and forests only a short drive away from any city in West Virginia.

### Workplace extension

As outlined in the pathophysiology section, workplace stress is a primary contributor to insulin resistance due to glucocorticoid signaling. Westfall encourages researchers to meet participants in settings where they are most likely to benefit [78]. Workplace diabetes prevention programs will be admittedly more challenging, but may offer the best strategy for long-lasting behavior changes. If these programs are implemented during lunch periods, they may be more likely to be sustained. Meeting participants in the workplace can provide lasting changes implemented through a community-focused adjustment in environment. One of the potential limitations for community-based programs outlined by Merzel and D'Afflitti is poor study design and evaluation [79]. In order to counteract this limitation, it will be essential to engage managers and workplace directors to get their buy-in. One way to do this will be to share relevant data regarding fewer missed workdays among healthier employees who have higher productivity, and are generally more positive [80].

Another potential challenge is comfort level of the participants. Places of worship such as churches, synagogues, temples and mosques as well as community centers, such as the local YMCA/YWCA, offer safe and constructive learning environments. Working environments are typically stressful and filled with time constraints. The lack of comfort is offset by practicality. If participants can learn to implement changes in stressful environments, the changes will be more likely to endure over time. Adam pioneered a stress reduction component into a community-based prevention program, which was very successful and associated with significant improvement in health outcomes [81]. Work environments have better adoption of community-initiated programs if the social norms are focused on healthy living [82]. In other words, as individuals begin to increase habits of healthy eating and physical activity others will begin to do the same.

## Conclusions

The metabolic syndrome poses a unique problem for public health professionals as the prevalence continues to rise. Insulin resistance is the primary underlying pathophysiologic change associated with the metabolic syndrome. Diabetes, obesity, and cardiovascular disease prevention through a CBPR approach offers to be the most cost-effective solution for widespread community change. In this review, metabolic syndrome prevention was discussed in the broader context of the West Virginia Diabetes Prevention and Management program. The program incorporates important aspects of participatory research such as participant feedback, health coaches' weekly feedback and calls to participants, a localized church setting, use of weekly food logs and pedometers to track dietary habits and physical activity, and changes in behavioral, anthropometric and clinical outcomes over the program period. These aspects were part of a broader approach that contains elements of participatory research, community engagement, and health outcomes research. Going forward it will be critical to engage participants in evaluation and dissemination of the data. To maximize sustainability, it will be important to advocate for increased funding and to role out programs in practical environments such as the workplace. In future programs, it will be important to identify factors that contribute to successful implementation of lifestyle modifications in real-world community settings. Overall CBPR programs that are focused on diabetes prevention or prevention of chronic diseases can produce substantial results if applied vigorously across the nation.
